# Can or should we try to predict preterm white matter injury?

**DOI:** 10.1038/s41390-024-03524-6

**Published:** 2024-08-22

**Authors:** Simerdeep K. Dhillon, Joanne O. Davidson, Justin M. Dean, Laura Bennet, Alistair J. Gunn

**Affiliations:** Department of Physiology, The University of Auckland, Auckland, New Zealand

Multiple advances in neonatal medicine such as antenatal corticosteroids and non-invasive ventilation support have improved survival after preterm birth,^1^ particularly for infants born at the threshold of viability (< 24 weeks of completed gestation).^2^ Despite this steady improvement in survival, the risk of adverse neurodevelopmental outcomes has not improved in proportion.^3^ Early identification of extremely preterm infants at risk of brain injury remains challenging.^4^ Magnetic resonance imaging (MRI) is typically undertaken at term-equivalent age, and is expensive and not available to all centers.^5^ In principle, early prediction of outcomes would be helpful to inform both healthcare professionals and parents,^6^ for example by using models based on clinical variables.^4,7,8^

In this issue, Song et al. report a study of 7566 extremely preterm infants (gestation age < 28 weeks) born between 2010 and 2019 across 68 clinical centers in China to identify the prenatal and postnatal predictors of white matter injury and develop a prediction tool (nomogram) for early identification of the high-risk infants.^9^ They found that a nomogram incorporating multiple gestations, premature rupture of membranes, chorioamnionitis, prenatal glucocorticoids, 1 minute Apgar score, and hypertensive disorder complicating pregnancy as prenatal factors predicted white matter injury on MRI during neonatal intensive care with an area under the curve of 0.799 in the external validation group. Postnatal factors, including days of mechanical ventilation, shock, grade III-IV intraventricular hemorrhage, patent ductus arteriosus ligation, septicemia, stage II-III necrotizing enterocolitis, and hypothermia, predicted MRI-defined white matter injury with an area under the curve of 0.823.

The strengths of this study include that the study was very large, and the online toolkit is easy-to-access and can examine cumulative effect of multiple prenatal or postnatal factors, and show the relative contribution of each variable to the risk of white matter injury. In practice, we submit that predicting white matter injury on MRI per se would only have limited clinical utility, as it does not provide individualized prediction of neurodevelopmental outcomes per se. Rather, the value of this or similar approaches is likely to be to help recruit a high risk population of preterm infants for clinical trials of potential therapies for evolving brain injury, instead of offering treatment to all extremely preterm infants as is often done at present.^10,11^

The reader should consider some limitations of the work. The type, location and timing of white matter injury on MRI were not assessed. There is considerable evidence that the severity and location of white matter injury differentially predict motor and cognitive outcomes in extremely preterm infants.^12^ Further, MRI was not standardized to term-equivalent age, but was performed when infants reached a body weight of 1800 g or before discharge. It is unclear if there were differences in the age at which these early MRIs were performed. The authors used the least absolute shrinkage and selection operator and multivariable logistic regression to identify predictors. Potentially, these linear methods may not fully encapsulate non-linear relationships and interactions between the predictors and outcomes.^6^

This study also examined prenatal and postnatal factors as separate risk profiles, but the interaction between these factors was not assessed. For example, prenatal factors such as chorioamnionitis and antenatal glucocorticoid treatment could potentially affect postnatal respiratory outcomes and the need for mechanical ventilation, and these factors can have synergistic effects on brain inflammation and injury.^13,14^ It will be important in the future, to incorporate these complex interactions. Next, despite the large sample size, it is reasonable to consider that the study was conducted on babies born in tertiary centers in China who received relatively homogenous perinatal care. The predictive value of the model or refinements needs to be validated across heterogeneous population groups and countries.

Next, the study population included only a small number of infants born before 26 weeks of completed gestation (median 27; interquartile range 26−27.4 weeks), which limits application of the prediction tool to infants at the threshold of viability, who are at the highest risk of perinatal complications and brain injury. Gestational age is not included as an input predictor in the online tool. Finally, the study was conducted in a retrospective cohort of infants born over a 10 year period, up to 2019. This predictive model may need to be updated as perinatal management, patient populations, and outcomes change over time. Therefore, continuous data inputs will be required to adjust for such changes.

In summary, the findings of Song et al. are a promising first step, but at present the value of assessing the risk of white matter injury is primarily for recruitment for treatment studies. It will be important to broaden the scope of these models to assess the prediction of long-term neurodevelopmental outcomes, to assesses non-linear interactions between perinatal variables, and to determine if the risk factors are same for infants born closer to the limits of viability.
